# Evaluation of the safety and immunogenicity in United Kingdom laboratory workers of a combined *Haemophilus influenzae* type b and meningococcal capsular group C conjugate vaccine

**DOI:** 10.1186/1745-6673-9-26

**Published:** 2014-07-16

**Authors:** Jamie Findlow, Helen Findlow, Sarah Frankland, Ann Holland, Daniel Holme, Emma Newton, Jo Southern, Pauline Waight, Ed Kaczmarski, Elizabeth Miller, Ray Borrow

**Affiliations:** 1 Public Health England, Public Health Laboratory, Manchester, Manchester Medical Microbiology Partnership, PO Box 209, Clinical Sciences Building II, Manchester Royal Infirmary, Manchester M13 9WZ, UK; 2 Immunisation Department, Health Protection Services, Public Health England, Colindale, London NW9 5EQ, UK; 3 University of Manchester, Inflammation Sciences Research Group, School of Translational Medicine, Stopford Building, Manchester M13 9PL, UK

**Keywords:** Meningococcal, *Haemophilus influenzae* type b, Tetanus, Vaccine, Laboratory workers, Occupational immunisation

## Abstract

**Background:**

Although a combined *Haemophilus influenzae* type b (Hib)/meningococcal capsular group C (MenC) conjugate vaccine with a tetanus toxoid carrier protein (Hib/MenC-TT) is not licensed for use in those above 2 years of age due to lack of data on safety and efficacy, certain patient groups at high risk of MenC and/or Hib disease are recommended to receive it. Laboratory workers working with Hib and/or MenC cultures may be at a potentially increased risk of acquiring infectious diseases and vaccination is therefore an important safety consideration. We undertook a clinical trial to investigate the safety and immunogenicity of Hib/MenC-TT vaccine in this cohort.

**Methods:**

A total of 33 subjects were recruited to the trial, all of whom were vaccinated. Serology was completed on samples taken at baseline and four weeks following vaccination to determine MenC specific IgG, MenC serum bactericidal antibody (SBA), anti-Hib polyribosylribitol phosphate (PRP) IgG and anti-tetanus toxoid IgG responses.

**Results:**

At baseline, high proportions of subjects had protective antibody concentrations against MenC, Hib and tetanus due to previous vaccination and/or natural exposure. Vaccination induced > 3, 10 and 220 fold increases in geometric mean concentrations for MenC SBA, anti-tetanus toxoid IgG and anti-Hib PRP IgG, respectively. Following vaccination, 97% of subjects had putative protective SBA titres ≥ 8, 100% had short term protective anti-Hib PRP IgG concentrations ≥ 0.15 μg/mL and 97% had protective anti-tetanus toxoid concentrations ≥ 0.1 IU/mL. No safety concerns were reported with minor local reactions being reported by 21% of subjects.

**Conclusions:**

Immunological responses determined in this trial are likely a combination of primary and secondary responses due to previous vaccination and natural exposure. Subjects were a representative cross-section of laboratory workers, enabling us to conclude that a single dose of Hib/MenC-TT was safe and immunogenic in healthy adults providing the evidence that this vaccine may be used for providing protection in an occupational setting.

## Background

Glycoconjugate vaccines to provide protection against *Haemophilus influenzae* type b (Hib) and *Neisseria meningitidis* capsular group C (MenC) were implemented into the UK immunisation schedule in 1992 and 1999, respectively [1,2]. The Hib vaccine was extremely effective in the targeted age group reducing invasive disease in England and Wales from almost 500 cases per year to 20 cases, two years following implementation [1]. The MenC vaccine was similarly successful and reduced disease incidence by 86.7% in the targeted age groups, also within two years of implementation [3]. Despite the success of these vaccines, cases in the UK general population still occur from which live isolates are initially cultured in local microbiology laboratories prior to transfer to reference laboratories at Public Health England (PHE). Transmission of Hib and MenC is achieved via the aerosol/respiratory route and as laboratory staff handle live cultures they can be considered to have a potential occupational exposure. Use of live cultures is not restricted to clinical and reference laboratories as there are many additional laboratories undertaking research. This results in a significant population of laboratory workers with a potential exposure risk including those undertaking functional immunoassays such as the MenC and Hib serum bactericidal antibody (SBA) assay to evaluate vaccine responses.

The potential risk was confirmed in an analysis conducted in the UK which determined laboratory workers to have a 184-fold increased risk of meningococcal disease compared to the general population [4]. This supports the requirement for employers to provide protection wherever possible to laboratory staff, with a potential occupational exposure to infectious disease [5]. Protection from acquisition and disease in the laboratory should primarily rely on physical control measures, however occupational vaccination is an important final form of defence. This is highlighted by a number of reports of potentially vaccine-preventable meningococcal cases in laboratory staff [6-10].

Occupational vaccination in the UK against meningococcal disease over the last decade has been generally achieved using monovalent MenC conjugate (MCC), A and C bivalent polysaccharide and quadrivalent A, C, Y and W vaccines initially in the form of polysaccharide formulations which have now been superseded by conjugate products [11]. Vaccination against Hib has been more problematic as the only available vaccines are combination vaccines designed for infant immunisation. In 2005, Menitorix® a combined Hib/MenC conjugate vaccine with a tetanus toxoid (TT) carrier protein was licensed in Europe and incorporated into the UK immunisation schedule from September 2006 as a 12 month booster vaccination [1]. The vaccine is also licensed for primary vaccination in infants from 2 months up to 12 months of age as a three dose course given with an interval of at least 1 month between doses. Although not licensed or generally for use in children above 2 years of age due to lack of data on safety and efficacy, it is recommended in certain patient groups to reduce the number of immunisations required [11]. Children and adults with asplenia or splenic dysfunction, may have a suboptimal response to MCC vaccine [12] and are recommended to receive a single dose of Hib/MenC-TT followed one month later by a single dose of a quadrivalent meningococcal A, C, Y and W conjugate vaccine [11].

The availability of Hib/MenC-TT vaccine provided the opportunity to offer vaccination to laboratory staff who routinely work with live Hib and/or MenC cultures at the Manchester Medical Microbiology Partnership (MMMP). We therefore undertook a clinical trial to evaluate the immunogenicity and safety of a single dose of Hib/MenC-TT vaccine in staff at a potential occupational exposure to Hib and/or MenC.

## Methods

### Study population and schedule

Enrolment into this single dose Hib/MenC-TT vaccine trial was open to adult (≥18 years of age) laboratory staff from the MMMP who were considered to be at potential occupational exposure to Hib and/or MenC. Potential occupational exposure was defined as routine handling of live Hib and/or MenC cultures which included both scientific staff from the PHE Meningococcal Reference Unit and the Vaccine Evaluation Unit (VEU) and porters/autoclave staff. At the time of enrolment this incorporated a greater range of staff than were at that time required to receive occupational meningococcal vaccination which was limited to scientific staff. Inclusion criteria ensured participants had no contraindications to vaccination as specified in the “Green Book” [11], written informed consent and availability for at least the initial trial (vaccination) visit. To allow the vaccine to be provided to as many laboratory staff as possible, exclusion criteria were limited to known or suspected pregnancy.

The non-adjuvanted Hib/MenC-TT vaccine (0.5 mL dose containing 5 μg of Hib polysaccharide (polyribosylribitol phosphate (PRP)) conjugated to 12.5 μg of TT and 5 μg of MenC polysaccharide conjugated to 5 μg of TT (GlaxoSmithKline, UK), from a single lot (Batch number A76CA008A), was administered in the deltoid muscle of the non-dominant arm. Vaccination was deferred if the oral temperature measured was >38°C, or if there was acute illness on the day of vaccination. Blood samples were taken before and 4 weeks following vaccination. Subjects were asked to provide details of any medication taken at enrolment and throughout the follow up period and meningococcal vaccine histories were obtained from the Occupational Health Department.

### Serology

Serum samples were assayed in the VEU, using a multiplexed fluorescent bead assay to quantify IgG antibody concentrations to MenC, Hib PRP and tetanus toxoid, based upon previously published methodology [13,14]. Functional antibody activity against MenC was determined in the SBA assay as previously described [15]. The target strain in the SBA assay was C11 (C:16:P1.7-1,1) and the complement source was baby rabbit sera (Pel-Freeze Incorporated, Rodgerson, AZ, USA) (rSBA). rSBA titres were expressed as the reciprocal of the final serum dilution giving ≥ 50% killing at 60 minutes. For computational purposes, rSBA titres lower than the serum starting dilution of 4 were assigned a value of 2.

### Correlates of protection

Immune correlates indicative of protection have been established for Hib, MenC and tetanus [16-18]. Hib PRP IgG concentrations ≥0.15 μg/mL and ≥ 1.00 μg/mL are considered the putative short term and long term protective levels, respectively [17]. For MenC, a rSBA titre of ≥ 8, four weeks after vaccination was shown to predict short term clinical protection against MenC disease in the UK [16] while a rSBA titre ≥ 128 reliably predicted a SBA titre using human complement of ≥ 4 [19], the accepted hSBA correlate of protection [20,21]. For tetanus, anti-toxoid levels < 0.1 IU/mL denote susceptibility and levels ≥ 1.0 IU/mL are considered as providing long term protection [18,22].

### Safety

Subjects were monitored for 30 minutes following vaccination and then completed a daily health diary for seven days recording any local reactions (erythema, swelling, tenderness) and measurements if present. Oral temperature was also recorded as well as any visits to a doctor, hospital or general practitioner or any medication taken. A vaccine research nurse followed up subjects by telephone 24 hours following vaccination to enquire about any reactions or problems.

### Analyses

At each time point the proportions of subjects achieving rSBA titres ≥ 8 and ≥ 128, anti-PRP IgG antibody concentrations ≥ 0.15 μg/mL and ≥ 1.00 μg/mL and anti-tetanus toxoid IgG antibody concentrations ≥ 0.10 IU/mL and ≥ 1.00 IU/mL were calculated. MenC rSBA geometric mean titres (GMTs) and MenC-specific IgG, anti-PRP IgG and anti-tetanus toxoid IgG geometric mean concentrations (GMCs) with 95% confidence intervals (95% CI) were also calculated. Due to the small sample sizes, immunogenicity analysis did not include any formal statistical analysis.

### Governance

The trial was conducted in accordance with the 1996 International Conference on Harmonisation Good Clinical Practice (ICH GCP) guidelines, the 2000 Declaration of Helsinki and the 2004 EU Clinical Trial Directive. A favorable opinion was given by the UK Medicines and Healthcare products Regulatory Agency (MHRA) and the National Research Ethics Service, Northern and Yorkshire Research Ethics Committee. The EudraCT number was 2006-004302-74 and the trial was registered on the public website, http://www.clinicaltrials.gov under the identifier: NCT00503165.

## Results

**Figure 1:**
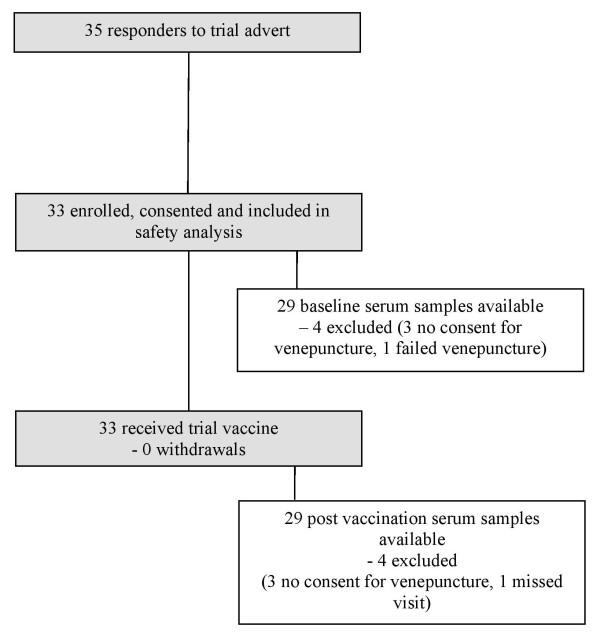
Participant flow chart.

A total of 35 staff responded to trial advertisements of which 33 were available for the first visit and were enrolled and vaccinated (Figure 1). Women accounted for 61% (20/33) of those enrolled reflecting the higher proportion of women laboratory staff within the eligible recruitment cohort. At enrolment the mean age of subjects was 30 years 2 months (range, 22 years 5 months to 59 years 10 months). Of the 33 subjects vaccinated, blood samples were obtained for 29 subjects at both time points. Three subjects did not consent to venepuncture but were still enrolled and vaccinated as the investigators’ principal aim was to afford potential benefit of vaccination to as many staff as possible. The trial was undertaken between July and September 2007 and the mean number of days between the baseline blood draw/vaccination and blood draw following vaccination sample was 31 days (range 18–64 days; median 28 days). Telephone follow-up at 24 hours was completed for all participants and diaries were completed and returned by 29 subjects.

### Immunogenicity

Prior to vaccination, 79% and 66% of subjects had putative protective MenC rSBA titres ≥ 8 and at the higher cut off of ≥ 128, respectively (Table 1). Following vaccination, a 3 fold increase in MenC rSBA GMT was determined whilst MenC-specific IgG GMC only increased 1.5 fold (Table 1). Proportions of subjects with rSBA titres above the two cut offs increased from the initially high background levels with all but one subject achieving an rSBA titre ≥ 8 following vaccination (Table 1).

**Table 1:** Proportions of subjects with MenC serum bactericidal antibody titres ≥8/≥128, MenC serum bactericidal antibody geometric mean titres with 95% confidence intervals, anti-MenC IgG geometric mean concentration with 95% confidence intervals, anti-PRP Hib IgG geometric mean concentration with 95% confidence intervals and proportions of subjects with anti-PRP Hib IgG ≥ 0.15/≥1.00 μg/mL before and following vaccination with Hib/MenC-TT

	**Baseline**	**Following vaccination**
rSBA GMT (95% CI)	151.2 (52.2 – 437.9)	454.3 (200.9 – 1027.3)
N (%) with rSBA titres ≥8	23/29 (79%)	28/29 (97%)
N (%) with rSBA titres ≥128	19/29 (66%)	24/29 (83%)
MenC IgG GMC (95% CI) μg/mL	4.3 (2.1 – 8.8)	6.6 (3.3 – 13.1)
N (%) with anti-PRP Hib IgG concentrations ≥ 0.15 μg/mL	18/29 (62%)	29/29 (100%)
N (%) with anti-PRP Hib IgG concentrations ≥ 1.00 μg/mL	9/29 (31%)	28/29 (97%)
Anti-PRP Hib IgG GMC (95% CI) μg/mL	0.23 (0.09 – 0.60)	50.69 (24.28 – 105.84)

For Hib at baseline, high proportions of subjects (62%) had anti-PRP IgG ≥ 0.15 μg/mL, the putative short term protective concentration while a lower proportion (31%) had putative long term protective concentrations ≥ 1.0 μg/mL (Table 1). Following vaccination, a > 200 fold increase in anti-PRP IgG GMC was determined which resulted in all subjects achieving anti-PRP concentrations ≥ 0.15 μg/mL and all but one achieving concentrations ≥ 1.0 μg/mL (Table 1).

Anti-tetanus toxoid IgG serology undertaken to determine responses induced by the carrier protein indicated a > 10 fold increase in IgG GMC following vaccination in comparison to baseline levels (Table 2). This resulted in all but one subject achieving anti-tetanus toxoid IgG concentrations ≥ 0.10 IU/mL and ≥ 1.0 IU/mL compared to 90% and 45% at baseline, respectively (Table 2).

**Table 2:** Proportions of subjects with anti-tetanus toxoid IgG antibody concentrations ≥ 0.10/1.0 IU/mL and geometric mean concentrations with 95% confidence intervals at baseline and following vaccination with Hib/MenC-TT

	**Baseline**	**Following vaccination**
N (%) with anti-tetanus toxoid IgG concentrations ≥ 0.10 IU/mL	26/29 (90%)	28/29 (97%)
N (%) with anti-tetanus toxoid IgG concentrations ≥ 1.00 IU/mL	13/29 (45%)	28/29 (97%)
Anti-tetanus toxoid GMC (95% CI) IU/mL	0.77 (0.47 – 1.27)	8.20 (4.65 – 14.46)

### Safety

Concomitant medication was reported by 11 (33%) participants. Two participants (6%) took over the counter tablets – one took Ibuprofen for an exercise injury on day 0 of the trial, and the other took paracetemol on day 8 of the trial for a raised temperature and nasal congestion. Nine participants (27%) took medication for chronic conditions during the trial, which the trial medic assessed as unrelated to trial treatment.

From the 29 diaries returned, six participants (21%) reported local reactions following vaccination, of which tenderness was most frequently reported (Table 3). Five participants (17%) reported systemic symptoms (Table 3). Participants reporting hay fever, hypertension and nasal congestion/raised temperature were medicated as above. In one participant an episode of dizziness and nausea commenced approximately 8 hours following vaccination and subsided after 48 hours. The individuals with hypertension and hay fever consulted their GPs about these conditions. There were no visits to hospital, or Serious Adverse Events reported during the trial.

**Table 3:** Local and systemic reactions reported by subjects following vaccination

**Local reactions**	
Any	6/29 (21%)
Tenderness	4/29 (14%)
Erythema	2/29 (7%)
**Systemic reactions**	
Any	5/29 (17%)
Headache	1/29 (3%)
Nausea/dizziness	1/29 (3%)
Hypertension	1/29 (3%)
Nasal congestion/raised temperature	1/29 (3%)
Hay fever	1/29 (3%)

## Discussion

To our knowledge this trial is the first report of the safety and immunogenicity of a Hib/MenC-TT combination vaccine in healthy adults which to date, has only been approved for children from 2 months up to 2 years of age. The vaccine was safe and immunogenic in the trial population indicating that it is suitable for use in an occupational setting to provide protection to laboratory staff which may have a potential occupational exposure to Hib and/or MenC.

Investigation of the immunogenicity of the MenC vaccine component was complicated as trial subjects were laboratory workers, who had previously received meningococcal vaccination. Consequently, 79% of subjects had baseline MenC rSBA titres ≥ 8 which is at least double, than previously reported in the UK general population ≥ 25 years of age in 2009 [23]. At enrolment, subjects with prior vaccination against MenC had been vaccinated with MCC vaccine either through the MCC catch-up campaign during 1999 to 2000 [2], a MCC staff trial [24] and/or through the Occupational Health Department with multivalent polysaccharide vaccines. Eight subjects had also received 3 doses of the “Norwegian” outer membrane vesicle (OMV) vaccine, MenBvac (Norwegian Institute of Public Health), in a trial in 2005/6 [25]. The OMV vaccine may have been responsible for elevated baseline rSBA titres due to induction of cross-reactive antibodies against subcapsular proteins on the MenC target strain. Of the six staff without putative protective MenC rSBA titres, three were not required to be vaccinated by the then current occupational vaccination program although two had received MCC vaccination seven years previously. The three other staff had received a quadrivalent polysaccharide vaccine three years previously and were two years away from the recommended five year re-vaccination point [11]. The clinical significance of SBA titres below the protective level is undetermined as while SBA titres ≥ than the protective level equate to protection from disease, a SBA titre < than the protective level does not necessarily indicate susceptibility [20,21].

Following vaccination, all but one subject achieved MenC rSBA titres ≥ 8 and the MenC rSBA GMT increased three fold from baseline levels. The rSBA response was mainly achieved in subjects with low baseline titres as the average individual fold change in rSBA titre following vaccination was 226.0 and 1.6 in subjects with baseline rSBA titres < 128 and ≥ 128, respectively (data not presented). This is in agreement with previous studies where negative correlations between baseline antibody concentrations and subsequent booster responses have been reported, including trials using Hib/MenC-TT as a booster [26].

The phenomenon of immunological hyporesponsiveness, characterised by decreased immunological responses upon repeated doses of MenC containing vaccines after initial MenC polysaccharide vaccination has been well documented in both adults [27-30] and younger children [31-33]. Two previous laboratory staff studies utilising MCC vaccine also demonstrated hyporesponsiveness in staff who had previously been vaccinated with meningococcal A and C bivalent polysaccharide vaccine in comparison to naïve controls [24,34]. Unfortunately, we were not able to make the same comparison in our own trial as all but one of the subjects had previously received meningococcal vaccination. Interestingly, these two trials reported greater increases in rSBA GMT and IgG GMC in both the naïve and the previously vaccinated groups than achieved in the trial we report here. It is difficult to determine the reason for this although the most apparent differences were that the previous trials used MCC as opposed to Hib/MenC-TT and many of the subjects in our trial had more complex previous meningococcal vaccination histories with high baseline immunity against MenC. In the future, any detrimental impact of prior polysaccharide vaccination should hopefully become less of an issue as meningococcal quadrivalent conjugate vaccines are now available and are recommended over polysaccharide vaccines for use in at risk-groups [11].

Despite none of the subjects reporting previous vaccination against Hib, 62% and 31% of subjects had baseline anti-Hib PRP IgG levels consistent with short and long term protection, respectively. These proportions were similar, although higher than those reported in the UK general population in 2009, whereby 57% and 21% of the 25–44 year age group were reported to have short and long term protective anti-Hib PRP IgG levels, respectively [35]. Following vaccination a > 200 fold rise in anti-PRP IgG GMC was measured, all subjects achieved short term protective antibody concentrations and all but one subject achieved an anti-PRP level ≥ 1.00 μg/mL.

Subjects also had baseline tetanus toxoid IgG levels similar to that in the UK adult general population in 2009 [36] with 10% and 45% of subjects at baseline and 17% and 44% of the UK general population with susceptible and long term protective antibody concentrations, respectively. Following vaccination a >10 fold rise in IgG GMT was achieved and all but one subject seroconverted to achieve IgG levels over 0.1 IU/mL. These data indicate that immune response to Hib/MenC-TT was not restricted to the two polysaccharide antigens and that the carrier protein is also able to induce a robust immune response in primed adults. Similar findings have previously been reported in meningococcal conjugate vaccines trials [37,38] and is an important finding as laboratory workers are also recommended to receive appropriate tetanus vaccination [11]. The ability of Hib/MenC-TT to boost tetanus antibody concentrations may negate the need to use an additional specific tetanus containing vaccine. This is of particular benefit as the unavailability of a monovalent tetanus vaccine in Europe, requires the use of a combination vaccine (Revaxis®, Sanofi Pasteur MSD) which in laboratory workers potentially unnecessarily contains diphtheria and inactivated poliovirus antigens.

Following Hib/MenC-TT, it was three different individuals who failed to achieve protective antibody levels against MenC, Hib and tetanus indicating that this was not due to a single individual/vaccination. Overall, the vaccine was well tolerated with a maximum of 21% of subjects reporting any systemic or local reactions of which they were generally minor and of short duration. This reactogenicity profile, is similar to that which would be predicted from previous Hib/MenC-TT trials undertaken in pediatric populations [39]. Unfortunately, it is not possible to compare reactogenicity to the one trial completed in older children as no safety data were reported [40].

## Conclusions

In conclusion, Hib/MenC-TT when administered as a single dose to healthy adults is safe and immunogenic. Due to subject’s previous meningococcal and tetanus vaccination in combination with probable natural exposure, immunological responses were likely to be a combination of primary and secondary responses. These are however, a representative sample of laboratory workers/healthy adults and therefore, provide evidence that this vaccine may be used in the occupational setting for providing protection against MenC and/or Hib. The vaccine also induced a robust response against the carrier protein, indicating that the vaccine can also boost protection against tetanus. The importance of these findings relates to the unavailability of monovalent conjugate Hib vaccines in Europe, necessitating the use of multivalent products of which Hib/MenC-TT has the lowest valency. These data reported here from healthy adults may also provide an insight into the immunogenicity and safety of Hib/MenC-TT in other at-risk populations, thus supporting the UK vaccination policy for vaccinating asplenic and hyposlpenic individuals [11].

## Competing interests

All authors are employees of PHE. JF, HF, SF, AH, DH, EN and RB undertake contract research on behalf of PHE for pharmaceutical companies, including GlaxoSmithKline, Novartis, Sanofi Pasteur MSD, Baxter and Pfizer. JF has acted as a consultant on behalf of PHE and has received travel assistance from GlaxoSmithKline, Novartis, Baxter and Pfizer. HF has acted as a consultant on behalf of PHE and has received travel assistance from Baxter.

## Authors’ contributions

RB and EM conceived the trial. JF, RB, EM and EK designed the trial and EK was the Chief Investigator and trial medic. JF, HF, SF, AH, DH and EN were responsible for completion of immunoassays. JF, JS, PW, EK and RB were responsible for trial coordination, maintenance of regulatory compliance and safety monitoring. All authors read and approved the final manuscript.
